# *Sarcopoterium spinosum* Inhibited the Development of Non-Alcoholic Steatosis and Steatohepatitis in Mice

**DOI:** 10.3390/nu11123044

**Published:** 2019-12-13

**Authors:** Ayala Wollman, Tehila Daniel, Tovit Rosenzweig

**Affiliations:** Departments of Molecular Biology and Nutrition Sciences, Ariel University, Ariel 40700, Israel

**Keywords:** non-alcoholic fatty liver disease, insulin resistance, natural agent, *Sarcopoterium spinosum*

## Abstract

Non-alcoholic fatty liver disease (NAFLD) is a comorbidity of obesity, which gradually develops from hepatic steatosis into steatohepatitis (NASH) and eventually even into fibrosis or hepatic carcinoma. To date, there has been no specific and effective treatment for NAFLD. *Sarcopoterium spinosum* extract (SSE) was found to improve insulin sensitivity. Recognizing the intimate link between insulin resistance and NAFLD, the aim of this study was to investigate the effectivity of SSE in the prevention and management of NAFLD at various severities. SSE was given to high-fat diet (HFD)-fed mice (steatosis model) or to mice given a Western diet (WD) in the short or long term (NASH prevention or treatment, respectively). SSE reduced body weight accumulation, improved glucose tolerance and insulin sensitivity and prevented the development of hepatic steatosis. SSE also blocked the progression of liver disease toward NASH in a dose-dependent manner. The expression of genes involved in lipid metabolism, inflammation, and antioxidant machinery was regulated by SSE in both models of steatosis and NASH development. However, SSE failed to reverse the hepatic damage in the advanced model of NASH. In summary, SSE might be considered as a botanical supplement for the prevention and treatment of hepatic steatosis, and for slowing the deterioration toward NASH.

## 1. Introduction

Non-alcoholic fatty liver disease (NAFLD) is an extremely common condition, affecting approximately 20% of adults in Western countries, with the highest prevalence of almost 75% affected subjects in the obese, diabetic population [1,2]. Based on this alarming statistic, NAFLD is considered as the leading cause of liver diseases [3]. The leading risk factors for NAFLD are obesity, type 2 diabetes (T2D), hypertension and dyslipidemia. All these factors are included in the metabolic syndrome. Accordingly, NAFLD is considered to be the hepatic manifestation of this syndrome [4,5].

Fatty liver disease is characterized by lipid accumulation in hepatocytes. Under the broad spectrum diagnosis of the disease is included a mild form, fatty liver, which manifests histologically by steatosis alone, without significant clinical symptoms. However, the accumulation of lipids is the first hit for hepatocyte injury, which sensitizes the liver to additional stressors, mainly oxidative stress (‘second hit’), which induce further hepatic injury [6,7]. Thus, in approximately 20% of cases, fatty liver may progress to a more severe form, steatohepatitis (non-alcoholic steatohepatitis (NASH) in the case of NAFLD), which is marked by the additional pathological features of lobar inflammation, hepatocellular ballooning and eventually tissue damage in the form of sinusoidal collagen formation representing initiation of liver fibrosis. Liver fibrosis may lead to cirrhosis, which involves risk of liver failure and hepatocellular carcinoma [4].

A close relationship exists between insulin resistance and hepatic steatosis. Insulin resistance is among the major mechanisms in the pathogenesis of hepatic steatosis [7] and in disease progression to NASH [8,9]. Most evidence supports the hypothesis that hepatic insulin resistance precedes hepatic steatosis [10,11,12]. The accumulated lipids lead to the synthesis of several lipid molecules, such as diacylglycerol and ceramides, which further worsen and stabilize the pathology of insulin resistance [13]. Therefore, targeting insulin resistance might be a preferred potential therapeutic strategy for NAFL/NASH disease [8,14].

*Sarcopoterium spinosum* (*S. spinosum*) is a chamaephyte of the *Rosaceae* family, which has been used in Bedouin folk medicine for its antidiabetic effect [15]. In our previous studies, we validated its glucose lowering properties, demonstrating an activation of the insulin signaling cascade, leading to an induction of glucose uptake in myotubes, hepatocytes and adipocytes [16,17]. These data were also supported by a set of in vivo experiments, demonstrating an improved glucose tolerance and insulin sensitivity in high-fat diet-fed mice and in genetic-prone type 2 diabetic mice (KK-Ay mice). We found an improved transmission of the insulin signaling cascade in skeletal muscle and liver of *S. spinosum*-treated mice. In addition, a moderate reduction in hepatic steatosis was demonstrated, indicating the potential therapeutic effect of this herbal product for NAFLD [18]. On the basis of these data, the aim of this study was to perform a comprehensive study on the effects of SSE on the development of steatosis and its progression toward NASH, utilizing diet-induced mice models of hepatic steatosis and NASH.

## 2. Materials and Methods

### 2.1. S. spinosum Extract Preparation

*Sarcopoterium spinosum* (L.) Spach. (Thorny burnet, local name: Natesh, Billan (Arabic), Sira Kotzanit (Hebrew)) was harvested from the wild in the area around Ariel University, in accordance with Israeli laws for biodiversity. A voucher specimen of the plant was deposited in the Israel National Herbarium at the Hebrew University of Jerusalem (No. HUJ 102531). Fresh *S. spinosum* roots (100 g) were cut into small pieces and boiled in 1 L of water for 30 min as described previously [18]. The extract was freeze-dried, and the resulting lyophilized powder was kept at room temperature till use.

### 2.2. Study Design

The study was carried out in accordance with the recommendations in the Guide for the Care and Use of Laboratory Animals of the National Institutes of Health. The Animal House in Ariel University operates in compliance with the rules and guidelines of the Israel Council for Research in Animals, based on the US NIH Guide for the Care and Use of Laboratory Animals. The protocols of the study were approved by the Committee on the Ethics of Animal Experiments of the University of Ariel (Permit Number: IL-148-01-18). The mice were housed in an animal laboratory with a controlled environment of 20–24 °C, 45–65% humidity, and a 12 h light/dark cycle. All efforts were made to minimize suffering.

#### 2.2.1. Steatosis Model

Experiments were performed utilizing the high-fat diet model of obesity (HFD, 60% of total calories derived from fatty acids, 36% saturated, 41% monounsaturated and 23% polyunsaturated; 18.4% from proteins, and 21.3% from carbohydrates, Teklad TD.06414). C57Bl/6 mice were purchased from Envigo (Jerusalem, Israel). The 6-week-old male mice were given either STD (18% of total calories derived from fat, 24% from proteins, and 58% from carbohydrates. Harlan, Teklad TD.2018) or HFD. At the age of 10 weeks, HFD-fed mice were separated into treatment groups as follows: HFD and HFD-fed mice supplemented with dried *S. spinosum* extract (SSE) at 3 different doses (30, 60 and 90 mg/day). All treatment groups included 8 mice. Body weight was measured once a week. At the age of 17 weeks, mice were anesthetized using ketamine + xylazine and euthanized by terminal bleeding followed by cervical dislocation. Blood was collected from the heart and serum was prepared. Serum insulin was measured by immunoassay, using an ELISA kit (Mercodia, Uppsala, Sweden). Liver was snap frozen in liquid nitrogen and preserved in −80 °C for later lipid extraction and mRNA analysis. Liver parts were saved in 4% paraformaldehyde for histological analyses.

#### 2.2.2. NASH Model

Non-alcoholic steatohepatitis (NASH) was induced by the feeding of Western diet (WD, 42% of total calories derived from fatty acids, 66% saturated, 30% monounsaturated, 4% polyunsaturated; 42.7% from carbohydrate, and 15.2% from protein, Teklad TD.88137) with the addition of fructose solution to drinking water (42 g/L, 55% fructose, 45% glucose).

##### Prevention of NASH

Mice were randomly divided into STD-fed (1 group, *n* = 8) or WD-fed (4 groups) for 4 weeks (from the age of six weeks until the age of 10 weeks). At the age of 10 weeks, WD-fed mice were separated into treatment groups (*n* = 8 each), as follows: WD and WD-fed mice supplemented with dried SSE at 3 different doses (30, 60 and 90 mg/day) for an additional 8 weeks.

##### Treatment of NASH

Mice were randomly divided into STD-fed (1 group, *n* = 8) or WD-fed (2 groups, *n* = 8 each) for 16 weeks of feeding. At the age of 22 weeks, WD-fed mice were separated into control and treatment groups (dried SSE, 90 mg/day) for an additional 6 weeks.

In both prevention and treatment protocol models, body weight was measured once a week. Mice were killed at the end of the intervention (20 or 28 weeks old in the prevention or treatment protocols, respectively). For that, mice were anesthetized by ketamin/xylazine and terminal bleeding was performed. Livers were perfused and saved for later histological, biochemical and molecular analyses. Serum was prepared and saved at −80 °C for later analyses of lipids and hepatic enzymes.

### 2.3. Glucose Tolerance Test (GTT)

An intraperitoneal glucose tolerance test (GTT) was performed at the age of 15 weeks (steatosis model) or 18 weeks (NASH prevention model). Glucose (1.5 mg/g body weight) was injected after 6 h fast, and blood glucose was determined from tail blood using the ACCU-CHEK Go glucometer (Roche, Mannheim, Germany).

### 2.4. Insulin Tolerance Test (ITT)

An insulin tolerance test (ITT) was performed in the HFD-fed mice model of steatosis at the age of 16 weeks following a 6 h fast. Glucose was measured following intraperitoneal insulin injection (0.75 U/kg).

### 2.5. Analysis of mRNA Expression by Real-Time PCR

Total RNA was extracted from the liver using TRI reagent (Molecular Research Center, Inc. Cincinnati, OH, USA) according to the manufacturer’s instructions. RNA (3 μg) was reverse transcribed by oligo dT priming (Stratascript 5.0 multi-temperature reverse transcriptase, Stratagene) according to the manufacturer’s instructionss. Real-time PCR was performed using SYBR Fast Universal Ready-Mix Kit (Kappa biosystems, Wilmington, MA, USA) by the MxPro QPCR instrument (Stratagene, San Diego, CA, USA). Primers for real time PCR reactions were synthesized by Sigma, Israel. Primer sequences were as shown in Table 1:

### 2.6. Measurement of Triglycerides (TG)

For the measurement of hepatic triglycerides, liver (100 mg) was homogenized in NP-40 solution (5%). The samples were twice heated to 80–100 °C for 5 min and cooled to room temperature. The samples were centrifuged for 2 min and the supernatant was used for triglycerides (TG) analysis using a Triglyceride Quantification Kit (Abcam, Cambridge, UK, AB 65336) according to the manufacturer’s instructionss.

### 2.7. Serum Cholesterol

HDL and LDL cholesterol were measured in a serum using a commercial kit according to the manufacturer’s instructions (Abcam, Cambridge, UK, AB 65390). Shortly, the serum was mixed with an equal volume of precipitation buffer, followed by 10 min incubation at room temperature and 10 min centrifugation at 2000× *g* for the separation of HDL cholesterol (supernatant) and LDL cholesterol (pellet). The pellet was resuspended with PBS. Cholesterol levels were measured in both fractions according to the manufacturer’s instructionss.

### 2.8. Hepatic Cholesterol Levels

Liver (10 mg) was homogenized in a solution of Chloroform:Isopropanol:NP-40 (7:11:0.1). The organic phase was collected, vacuum dried and resuspended in a Cholesterol Assay Buffer supplied by Abcam. Total cholesterol was measured by a Cholesterol/Cholesteryl Ester quantitation kit (Abcam, Cambridge, UK, AB 65359) according to the manufacturer’s instructions.

### 2.9. Serum ALT (Alanine Transaminase) and AST (Aspartate Transaminase)

Serum ALT and AST levels were measured in fresh samples using the alanine transaminase and aspartate aminotransferase activity assay kits (ab105134 and ab105130, respectively, Abcam, Cambridge, UK), respectively, according to the manufacturer’s instructions.

### 2.10. Histochemistry

Liver histology was evaluated at the end of the intervention periods; at the age of 17, 20 or 28 weeks in the steatosis model, NASH prevention and NASH treatment, respectively. Livers were perfused, isolated, fixed in 4% paraformaldehyde and embedded in paraffin. Consecutive 4 μm sections were cut and stained with hematoxylin and eosin (H&E). The presence of inflammation and steatosis score was blind evaluated by a pathologist. Scoring of liver sections was adapted from Liang W. et al. [19]. Evaluation was performed with an Olympus light microscope BX43, Olympus digital camera DP21 with Olympus cellSens Entry 1.13 software.

### 2.11. Statistical Analysis

Values are presented as the mean ± SEM. Statistical differences between the treatments and controls were tested by one-way or two-way analysis of variance (ANOVA), followed by Bonferroni’s post hoc testing, as appropriate. Analysis was performed using the GraphPad Prism 8 software. A difference of *p* ˂ 0.05 or less in the mean values was considered statistically significant.

## 3. Results

### 3.1. Effect of SSE on Hepatic Steatosis

**Figure 1:**
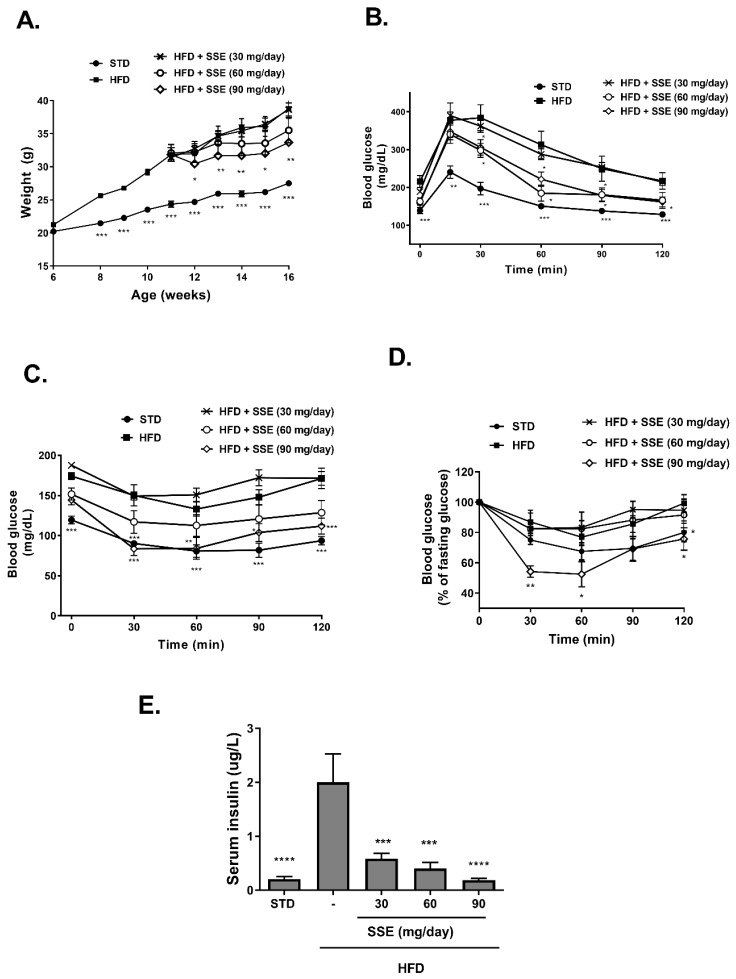
*Sarcopoterium spinosum* extract (SSE) improved glucose tolerance and insulin sensitivity in high-fat diet (HFD)-fed mice. STD or HFD-fed C57BL/6 mice were given SSE from the age of 10 weeks, as described in Methods. (**A**). Body weight was measured every week. (**B**). Glucose tolerance test (GTT) was performed at the age of 15 weeks as described in Methods. (**C**,**D**). Insulin tolerance test (ITT) was performed at the age of 16 weeks as described in Methods. The results are presented as absolute values (**C**) or as the percent of fasting glucose levels (**D**,**E**). Fasting serum insulin levels were measured at the age of 17 weeks. The results are presented as the mean ± SEM. * *p* < 0.05, ** *p* < 0.005, *** *p* < 0.0005, and **** *p* < 0.0001 by one-way (**E**) or two-way Anova (**A**–**D**) followed by Bonferroni’s post hoc test.

The effects of SSE on body weight and glucose homeostasis were followed. A significant increase in body weight was observed in all HFD-fed groups (Figure 1A). Food consumption and drinking habits were measured, demonstrating lower food consumption in all HFD-fed groups and no difference in drinking habits. Treatment by SSE consumed at the highest dose (90 mg/day) led to a lower body weight accumulation compared to their HFD-fed littermates. Fasting blood glucose and glucose disposal following intraperitoneal glucose load was impaired in HFD-fed mice (Figure 1B). These parameters were improved by SSE administration (60, 90 mg/day). Insulin resistance was developed in HFD-fed mice, as demonstrated by a reduced response to insulin load (Figure 1C,D presenting absolute and relative values of blood glucose, respectively). This resistance was corrected by SSE supplementation, leading to higher sensitivity to the hormone and lower levels of blood glucose. The effects of SSE on fasting serum insulin (Figure 1E) are in line with the ITT results; insulin levels were elevated by HFD feeding, while SSE negated this effect, leading to the normalization of fasting insulin levels. These results suggest a dose-dependent effect of SSE on glucose tolerance and insulin sensitivity in HFD-fed mice.

**Figure 2:**
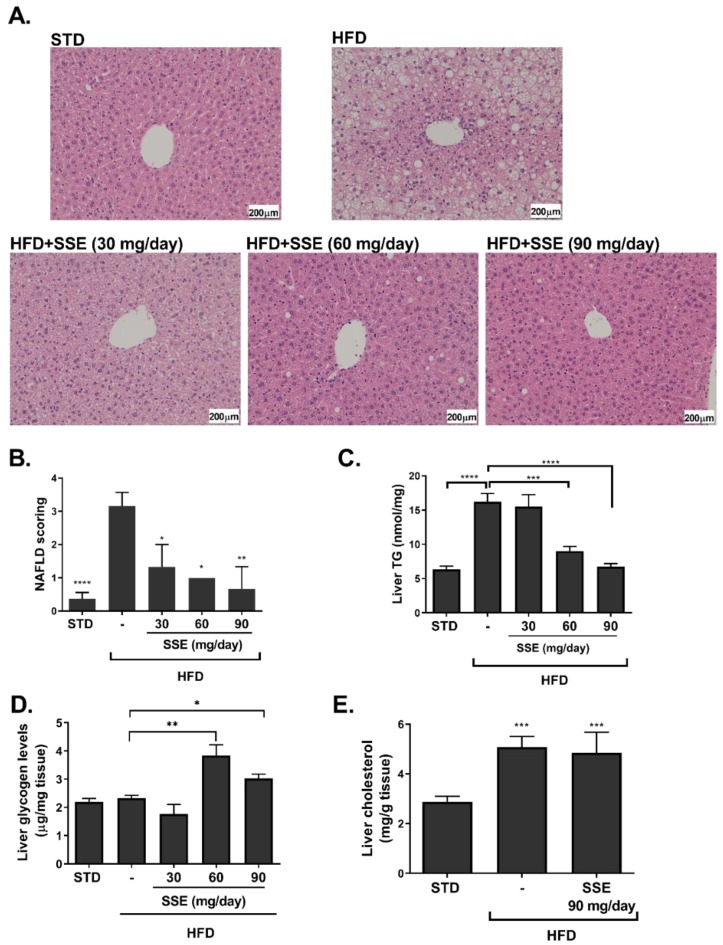
SSE reduced hepatic lipid content in HFD-fed mice. STD or HFD-fed C57BL/6 mice were given SSE from the age of 10 weeks, as described in Methods. Mice were killed at the age of 17 weeks and hematoxylin and eosin (H&E) staining of the livers was performed (**A**). The severity of non-alcoholic fatty liver disease (NAFLD) was evaluated by an independent pathologist as described in Methods (**B**). Hepatic triglycerides (**C**), glycogen (**D**) and total cholesterol (**E**) levels were measured. The results are presented as the mean ± SEM. * *p* < 0.05, ** *p* < 0.005 *** *p* < 0.0005, and **** *p* < 0.0001 by one-way Anova followed by Bonferroni’s post hoc test compared to STD-fed mice or as indicated.

HFD feeding induced an extensive hepatic steatosis, covering over 66% of the hepatic area. Lipid droplets were observed in almost all hepatocytes (Figure 2A). An improvement in liver steatosis was demonstrated in mice treated with 30 and 60 mg/day SSE, showing much smaller lipid droplets, and an almost complete normalization of liver morphology was found in HFD-fed mice treated by 90 mg/day SSE. A histological evaluation of the severity of hepatic steatosis, performed by an independent pathologist (Patho-Lab Diagnostics Ltd., Rehovot, Israel), revealed a reduction in NAFLD scoring in SSE-treated mice, in all doses given (Figure 2B). Biochemical measurement of hepatic TG level supported the histological data, demonstrating that TG levels were completely normalized by SSE treatment in a dose-dependent manner (Figure 2C). Hepatic glycogen and hepatic total cholesterol were also measured in STD, HFD and HFD-fed mice. Glycogen level was increased by SSE treatment (Figure 2D) while cholesterol level was higher in HFD-fed mice and was not affected by SSE supplementation (Figure 2E).

**Figure 3:**
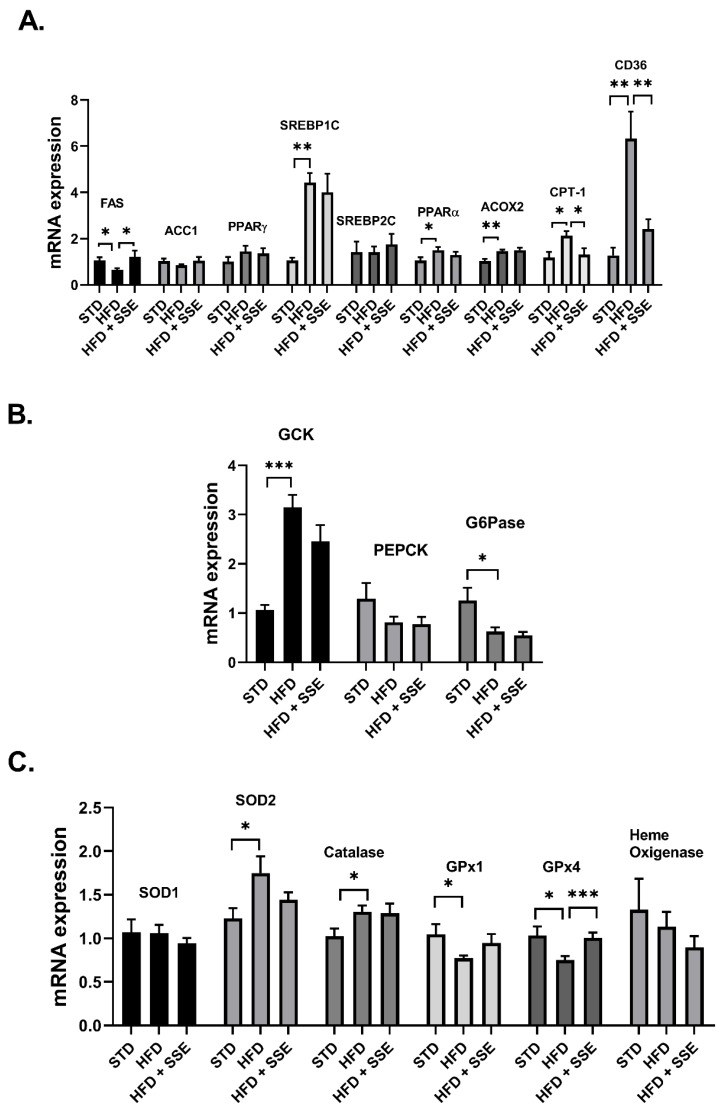
The effect of SSE on gene expression in livers of HFD-fed mice. STD or HFD-fed C57BL/6 mice were given SSE from the age of 10 weeks, as described in Methods. The mRNA expression of genes involved in lipid (**A**) and glucose (**B**) metabolism, and the antioxidant system (**C**) was measured. Results were normalized to the expression of the housekeeping gene, *RPS29*. The results are presented as the mean ΔΔCt ± SEM. * *p* < 0.05, ** *p* < 0.005, and *** *p* < 0.0005, compared to HFD by one-way Anova followed by Bonferroni’s post hoc test.

The expression of genes involved in lipid metabolism, inflammation and oxidative stress was measured in livers of STD, HFD and HFD + SSE (60 mg/day)-fed mice. SSE normalized the mRNA expression of *FAS*, *CPT-1* and *CD36*, encoding for proteins regulating lipid synthesis, oxidation and transport, respectively (Figure 3A). The mRNA expression of gluconeogenic and glycolytic enzymes was not affected by SSE treatment (Figure 3B). In view of the enzymatic antioxidant system, HFD feeding induced an increase in the mRNA expression of *SOD2* and Catalase and attenuated the expression of *GPx1* and *GPx4*. SSE normalized the expression of the latter antioxidant enzyme, while the expression levels of other enzymes measured were not affected by SSE (Figure 3C). The expression of genes involved in inflammation was not affected by HFD, either in the absence or presence of SSE.

### 3.2. SSE for the Prevention of NASH

**Figure 4:**
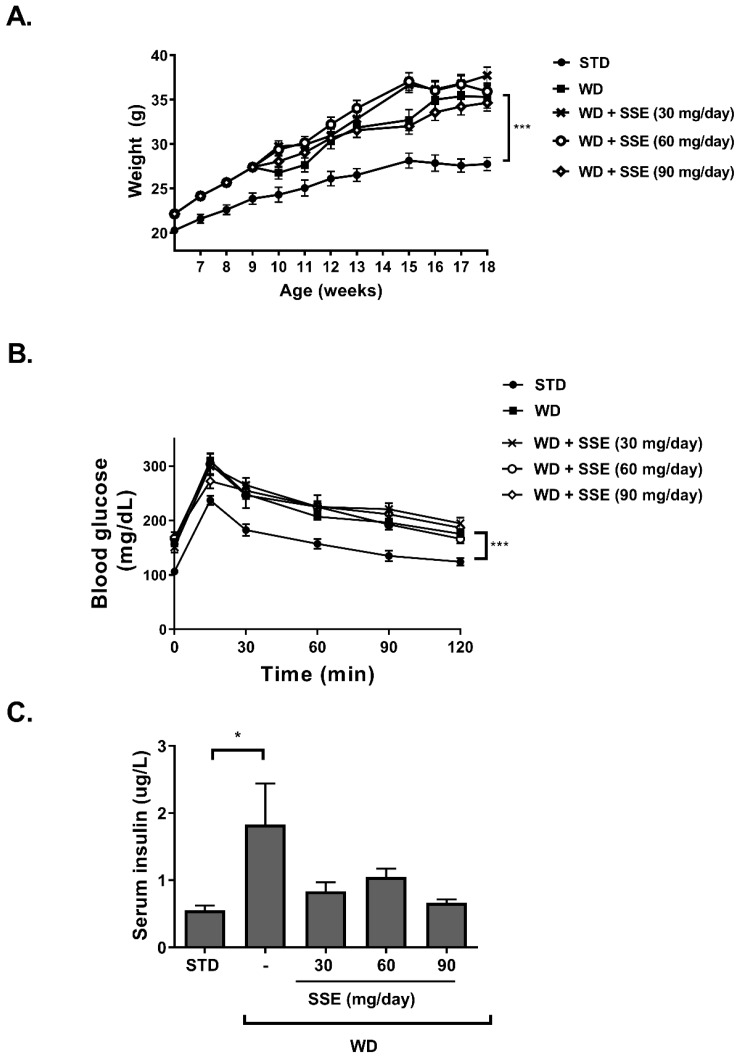
The effect of SSE on body weight and glucose tolerance in Western diet (WD)-fed mice. C57BL/6 mice were fed a STD or WD with or without SSE given from the age of 10 weeks, as described in Methods. (**A**). Body weight was measured every week. (**B**). Glucose tolerance test (GTT) was performed at the age of 18 weeks as described in Methods. (**C**). Fasting serum insulin levels were measured at the age of 20 weeks. The results are presented as the mean ± SEM. * *p* < 0.05 and *** *p* ˂ 0.0005 by one-way (**C**) or two-way Anova (**A**,**B**) compared to WD-fed mice, followed by Bonferroni’s post hoc test.

WD-fed C57BL/6 mice with access to fructose in drinking water (ad libitum) develop pathological characteristics of steatohepatitis [20]. NASH is characterized by hepatocellular steatosis with additional pathological features, such as hepatic inflammation and sinusoidal collagen formation representing initiation of liver fibrosis accompanied by a reduction in hepatic function. In order to characterize the beneficial properties of SSE on NASH, we followed its effect using two protocols. In the first NASH protocol, we analyzed the effect of SSE on the progression of the hepatic pathology toward NASH (a prevention protocol). Food consumption was reduced in all WD-fed groups and no difference was observed between all five groups in drinking habits. WD induced an increase in body weight accumulation, which was not affected by SSE consumption (Figure 4A). Fasting blood glucose and glucose tolerance were impaired by WD feeding, with lower severity compared to the metabolic disturbance induced by the HFD model (average fasting glucose of 159 mg/dL vs. 217 mg/dL in WD- and HFD-fed mice, respectively, and max glucose level was measured 30 min following a glucose load of 309 mg/dL vs. 416 mg/dL in WD- and HFD-fed mice, respectively). SSE did not affect fasting glucose or glucose disposal in WD-fed mice (Figure 4B).

Insulin load, performed as part of the insulin tolerance test at the age of 10 weeks old (in order to verify the development of metabolic alteration at the beginning of SSE intervention), induced severe hypoglycemia. Because of the hypoglycemic response, the insulin tolerance test was not performed in this model again, and thus no data are available on mice following SSE treatment. However, increased serum insulin was detected in WD-fed mice (Figure 4C) [21,22], which was corrected by SSE treatment.

**Figure 5:**
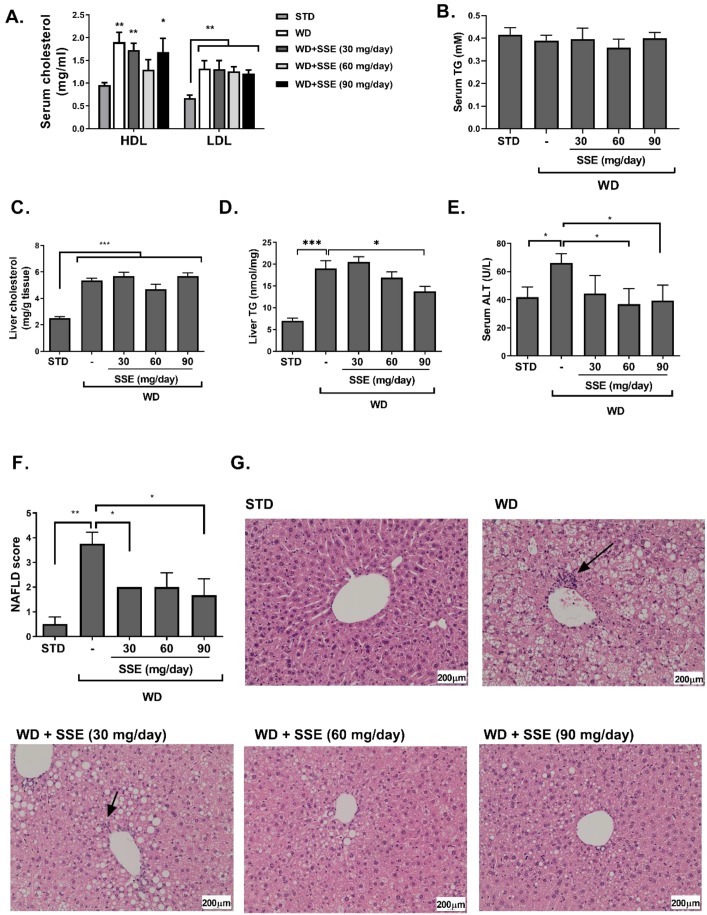
SSE inhibited the development of non-alcoholic steatohepatitis (NASH) in WD-fed mice. C57BL/6 mice were fed STD or WD with or without SSE given from the age of 10 weeks, as described in Methods. Mice were sacrificed at the age of 20 weeks. Serum LDL and HDL cholesterol (**A**), and serum triglycerides (**B**) were measured. In addition, total cholesterol (**C**) and triglyceride levels (**D**) were measured in the livers. (**E**) Serum alanine transaminase (ALT) was measured as described in Methods. H&E staining of livers was performed. (**F**) The severity of NAFLD was scaled as described in Methods. (**G**) Micrographs of H&E liver staining. Arrows point to foci of inflammation. Results are presented as the mean ± SEM. * *p* < 0.05, ** *p* < 0.005, and *** *p* < 0.005 by one-way Anova followed by Bonferroni’s post hoc test compared to STD or as indicated.

In order to characterize the effect of SSE on serum lipids, the levels of LDL and HDL cholesterol were measured. WD induced dyslipidemia, as demonstrated by the increased levels of both LDL and HDL cholesterol (Figure 5A), which were almost unaffected by SSE supplementation. Serum triglyceride (TG) was not affected by the diet or by SSE treatment (Figure 5B). Hepatic TG and cholesterol levels were both elevated in WD-fed mice. SSE (90 mg/day) reduced the severity of TG accumulation in the liver, while cholesterol levels were not affected (Figure 5C,D). Serum alanine transaminase (ALT) and aspartate transaminase (AST) levels were measured as an indication of liver damage. AST levels were not affected by the WD regime in all experimental groups. An increase in serum ALT was observed in WD-fed mice, which was completely corrected by SSE treatment, suggesting that SSE eliminates the hepatic damage induced by the diet (Figure 5E).

Histological evaluation of the severity of the disease in livers of WD-fed mice, with or without SSE treatment, clearly indicates lower NAFLD scores in treated mice (Figure 5F,G). While WD-fed mice developed a severe hepatic steatosis, covering over 66% of liver samples, SSE reduced the severity of steatosis to a level recognized as mild, covering 5–33% of liver samples, in all doses used in this study. In addition, foci of inflammation were detected in livers of WD-fed mice, but not in SSE-treated mice.

**Figure 6:**
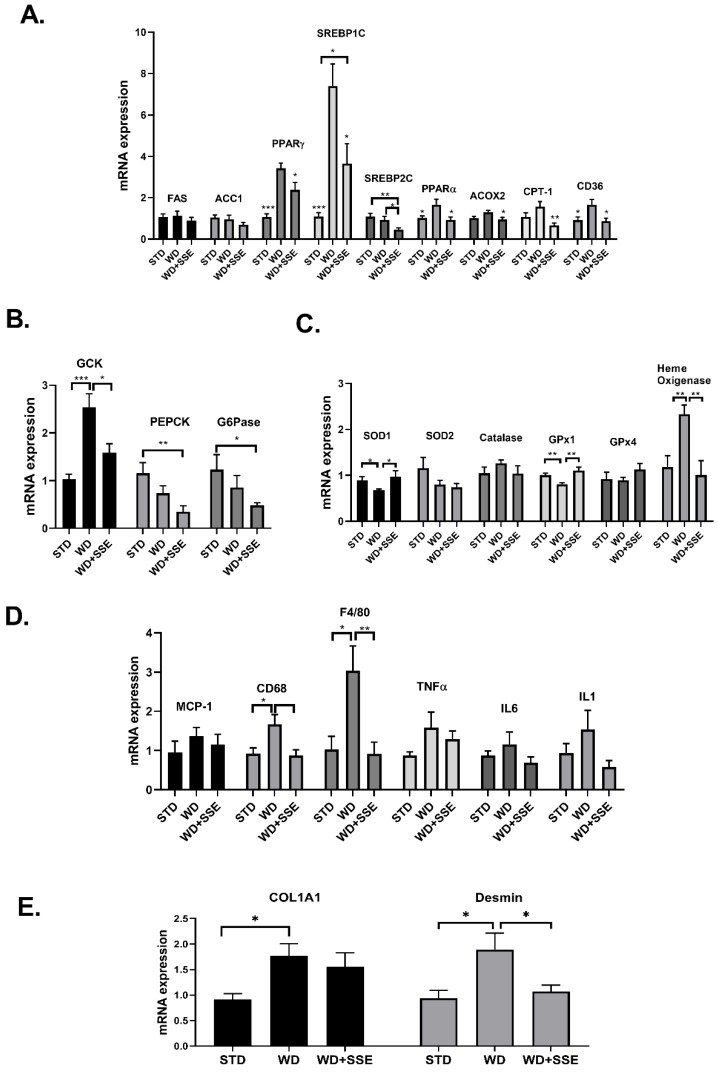
The effect of SSE on gene expression in livers of WD-fed mice. C57BL/6 mice were fed STD and WD with or without SSE (90 mg/day), given from the age of 10 weeks, as described in Methods. The mRNA expression of genes involved in lipid (**A**) and glucose (**B**) metabolism, the antioxidant system (**C**), inflammation (**D**) and fibrosis (**E**) was measured. Results were normalized to the expression of the housekeeping gene, *RPS29*. The results are presented as the mean ΔΔCt ± SEM. * *p* < 0.05, ** *p* < 0.005, and *** *p* < 0.0005 by one-way Anova followed by Bonferroni’s post hoc test compared to WD or as indicated.

The expression of genes encoding for enzymes involved in lipid metabolism revealed that while WD induced an alteration in the expression of most genes measured, SSE treatment normalized these disturbances (Figure 6A). Regarding glucose metabolism, SSE reduced the mRNA expression of *GcK* compared to WD-fed mice, and inhibited the expression of genes involved in gluconeogenesis, compared to STD-fed mice (Figure 6B). WD-induced alterations in the expression of antioxidant enzymes were reversed by SSE treatment to the expression level found in STD-fed mice (Figure 6C). Similarly, SSE negated the pro-inflammatory effect of WD, as determined by the lower mRNA expression of *F4/80* and *CD68*, with both markers of macrophages infiltration (Figure 6D). An elevation in the expression of *Col1A1* and *desmin*, markers of fibrotic response and hepatic stellate cell activation, respectively, was observed in WD-fed mice (Figure 6E). The increased expression of these genes, along with the increase in pro-inflammatory genes, support the development of mild NASH in this model, as reported before [23]. SSE treatment abrogated the induction of *desmin* expression, while not affecting the expression of *Col1a1*.

### 3.3. SSE for the Treatment of NASH

**Figure 7:**
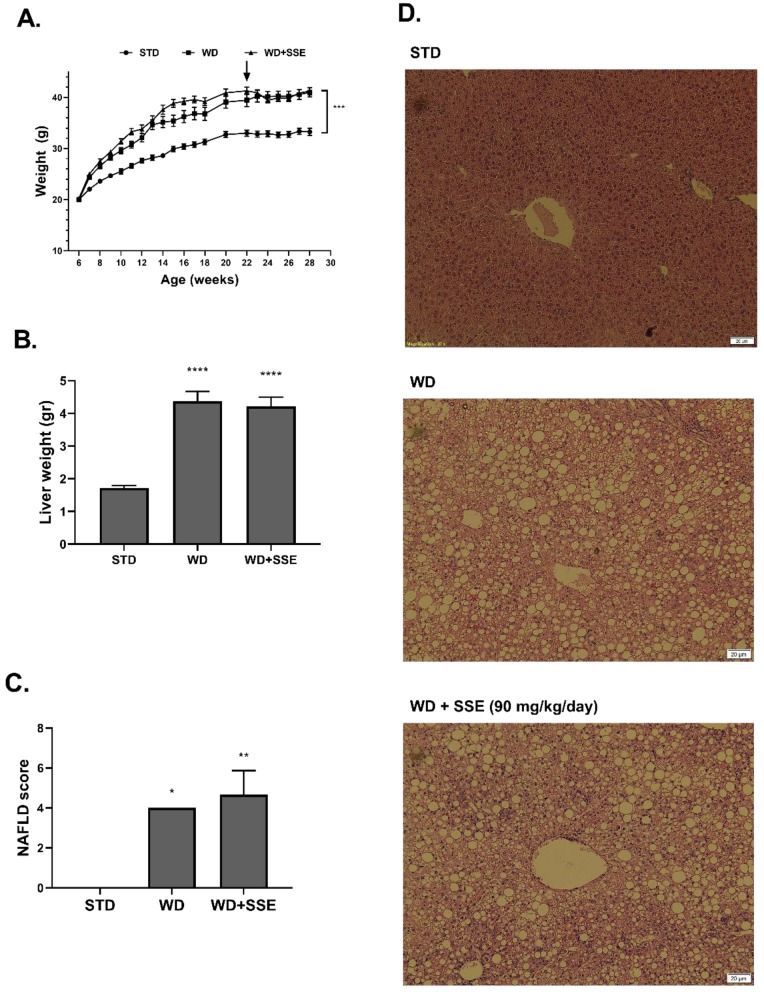
SSE failed to reduce the severity of advanced NASH. C57BL/6 mice were fed STD or WD from the age of 6 to 28 weeks, with SSE given in the last 6 weeks, as described in Methods. Body weight during intervention (**A**) and liver weight at the age of 28 weeks (**B**) were measured. (**C**). The severity of NAFLD was scaled as described in Methods. (**D**). Representing micrographs of H&E liver staining. The results are presented as the mean ± SEM, * *p* < 0.05, ** *p* ˂ 0.005, *** *p* ˂ 0.0005, and **** *p* ˂ 0.0001 by one-way Anova followed by Bonferroni’s post hoc test, compared to STD-fed mice.

In the second experimental setup, aimed to model the effectivity of SSE in reversing the NASH pathology (a treatment protocol), SSE was given to mice at the age of 22 weeks, following 16 weeks of a NASH-promoting diet. Body weight accumulation was higher in WD-fed compared to STD-fed mice, and SSE did not affect this parameter (Figure 7A). Similarly, SSE did not reduce the severity of liver pathology (Figure 7B–D).

## 4. Discussion

In this study, we investigated the potential benefits of SSE supplementation on the treatment of NAFLD with increasing levels of severity. HFD feeding was given to induce obesity, insulin resistance and simple hepatic steatosis. WD, with the addition of fructose to drinking water, resembles the “fast food” diet consumed in many Westernized countries, which is rich in saturated fatty acids and simple carbohydrates. This diet was used as a model of steatohepatitis [20]. SSE was most effective in the treatment of HFD-induced compared to WD-induced pathologies. HFD feeding is known to impair insulin sensitivity of target tissues, such as muscle and liver. It was found that an aberrant response to insulin is developed in the liver in little as 3 days of HFD feeding before the development of overt obesity [24,25]. This timeline points to insulin resistance as a major underlying disturbance, contributing to other HFD-associated disorders, which develop later, such as metabolic inflammation and fatty liver steatosis. Although the cause–effect relationship between hepatic lipid accumulation and insulin resistance is still open to debate, there is no doubt about the major role insulin resistance plays in the pathology of NAFLD [26]. Insulin resistance increases hepatic lipogenesis via several mechanisms. Systemic insulin resistance increased adipose tissue lipolysis, leading to an increased flux of FFAs, the substrate for lipogenesis, to the liver [27]. In addition, insulin resistance is paradoxically associated with an elevated rate of *de novo* lipogenesis. Despite the failure to suppress hepatic glucose production, an accelerated rate of lipid synthesis is observed in insulin-resistant patients [28]. This phenomenon suggests a selective insulin resistance of the liver, in which some arms of the insulin signaling pathway are insensitive to the hormone, while the lipogenic arm remains sensitive [29,30]. Although the mechanisms of this specific hepatic insulin resistance are as of yet uncovered, the clinical implication is that targeting insulin resistance is an important strategy in the prevention and treatment of hepatic steatosis.

Accordingly, the observations obtained in this study, demonstrating a significant improvement in insulin sensitivity in HFD-fed mice following SSE treatment, are promising. We demonstrated that SSE supplement abrogated the adverse effects of HFD on insulin sensitivity—responses to insulin load were normalized and hyperinsulinemia was abolished. This study supports our previous work, also indicating that SSE exerts an insulin sensitizing activity. We already demonstrated that SSE improved glucose tolerance and insulin sensitivity in a genetic model of T2D and in HFD-fed glucose-intolerant mice [18]. However, while in our previous studies SSE was given as a preventive protocol [16,17,18], in this study, we demonstrated a dose-dependent effect of SSE on glucose intolerance also when given after the development of the metabolic alteration. The mechanism mediating the effects of SSE on insulin sensitivity requires additional clarification, although we were able to show that in myotubes and adipocytes SSE activates both the metabolic and mitogenic arms of the insulin cascade [31]. These results suggest that the active compound/s act on an upstream component of the signal and has an insulin mimetic activity.

Here, we show that SSE eliminated HFD-induced hepatic steatosis, demonstrating almost a normal histology of the liver. Hepatic gene expression was affected by SSE, presumably as a result of the metabolic changes induced by the treatment. HFD increased the expression of genes involved in fatty acid uptake (*CD36*) and lipid oxidation (*PPARα*, *ACOX2*), while it inhibited the expression of genes encoding for enzymes of the lipid biosynthesis pathway (*FAS*, *ACC1*). This is in accordance with previous proteomic analysis performed in livers of HFD-fed mice, suggesting that HFD feeding activates a self-protective mechanism to counteract the excessive lipid load [32]. SSE opposed the HFD effect on the expression of some of these genes. SSE increased the expression of the gene encoding for *FAS*, while it decreased the expression of *CPT-1*, which plays a key role in fatty acid oxidation. As this pro-lipogenic profile of gene expression was accompanied by lower hepatic lipid accumulation in SSE-treated mice, we suggest that SSE improved the metabolic profile in such a way that the ‘self-protecting’ mechanism, activated by HFD, is not activated. We suggest that by improving insulin sensitivity, SSE negates the overload of lipids (the lower expression of *CD36*, which plays a role in lipid transport into the cells, supports this assumption) by a mechanism that should be further clarified. We also found that SSE reduced the severity of diet-induced obesity, although, as only a moderate effect was observed, it appears that the reduced body weight cannot account for the normalization of insulin sensitivity and hepatic steatosis induced by SSE. Recognizing the link between insulin sensitivity and the mechanisms regulating energy balance [33,34,35], it could be speculated that by combating insulin resistance SSE also affects energy balance. The effects of SSE on food intake and energy expenditure are currently under investigation.

The progression of NAFLD from simple steatosis to NASH is a complex process, with several diverse defective events contributing to the hepatic damage. While it is still hard to predict which patients diagnosed with simple hepatic steatosis will progress to NASH, the severity of insulin resistance is considered as a risk factor [36]. Among the adverse factors contributing to the progression of the disease are oxidative stress, dysfunctional adipose tissue, inflammation, ER stress and alterations in the microbiome. Each factor promotes the disease, and the integrated effect further intensifies the pathology [37]. Insulin resistance is implicated in some of these alterations, supporting the significant contribution of insulin resistance to the buildup of NASH pathology [38]. Thus, liver disease progressed from steatosis to NASH just like a snowball, with insulin resistance playing a role as both initiator and accelerator of this disease [39]. Accordingly, targeting insulin resistance is a promising strategy in order to fight steatosis and NASH [40]. We found that SSE reduced the progression of fatty liver toward NASH in a mild NASH model, induced by Western diet feeding. SSE reduced the severity of steatohepatitis in the liver and improved hepatic function, when administrated at the early stage of the disease, 4 weeks following the administration of the NASH-promoting diet. Similar to its effects on gene expression in HFD-fed mice, SSE also reversed some of the alterations in the expression of genes encoding for proteins involved in lipid metabolism. In addition, SSE normalized the mRNA expression of macrophage markers, suggesting that SSE prevents the pro-inflammatory effect of the diet, in accordance with our previous publication [41].

Hepatic steatosis is accompanied by increased oxidative stress, which is explained by several mechanisms leading to both higher rate of ROS generation and lower activity of the detoxifying enzymes [42]. As expected, an alteration in the mRNA expression of several antioxidant (AOX) enzymes was observed in HFD and WD-fed mice. SSE normalized the expression of most of the affected genes. We suggest that the normalization in the expression of these AOX genes is a secondary outcome of the reduced hepatic steatosis. However, we cannot exclude the possibility that, as with many other botanical preparations, SSE exerts a direct AOX activity in addition to other effects of the extract.

Although found to be effective in the treatment of hepatic steatosis and in the prevention of NASH development, SSE was ineffective in reversing the NASH-associated hepatic damage once it was already established. As liver fibrosis is the major factor responsible for liver dysfunction and for mortality associated with NASH, the failure of SSE to reverse such damage further emphasizes the importance of early diagnosis and treatment of NAFLD disease. The effectivity of a combined treatment including SSE fighting the basic underlying metabolic pathology with other drugs directed to fight other pathologies of the advanced NASH, i.e., inflammation, cell stress and fibrogenic remodeling, should be investigated.

In summary, this is the first comprehensive study demonstrating that SSE is beneficial for the treatment of insulin resistance and fatty liver as well as the prevention of hepatic deterioration toward steatohepatitis. The doses of lyophilized SSE used in this study, were similar to the dose recommended for drinking by traditional medicinal practitioners [43], giving an additional support for the traditional use of this plant for the treatment of diabetes, insulin resistance and related pathologies. In addition, this study motivated the search for active compound/s and additional research is still required in order to bring SSE to patients, either as a whole extract (botanical drug), or as a chemical drug of its active component (as of yet unidentified).

## 5. Patents

Patent application entitled “S. Spinosum extract for treating fatty liver disease” was submitted (Application WO-2019043700-A1).

## Figures and Tables

**Table 1:** Primer sequences.

Gene	Forward	Reverse
*ACC1*	CATGAACACCCAGAGCATTG	ATTTGTCGTAGTGGCCGTTC
*ACOX2*	AAGTGGCCAGGTTTCTGATG	TCTTGGTGTGGCGAGATACA
*CAT*	TGACAAAATGCTTCAGGGCC	CTGGTTGTCATGCATGCACA
*CD36*	AGCAGCTGCACCACATATCTAC	GGAACCAAACTGAGGAATGG
*CD68*	CCAACAAAACCAAGGTCCAG	TGTATTCCACCGCCATGTAG
*COL1A1*	GTGTTCCCTACTCAGCCGTC	TCCGTACTCGAACGGGAATC
*CPT-1*	GATGTGGACCTGCATTCCTT	TCCTTGTAATGTGCGAGCTG
*Desmin*	CAGAGGCTCAAGGCCAAACTA	AGGGATTCGATTCTGCGCTC
*FAS*	TTGCTGGCACTACAGAATGC	AACAGCCTCAGAGCGACAAT
*F4/80*	CTGTAACCGGATGGCAAACT	ATGGCCAAGGCAAGACATAC
*Gck*	GCAGAAGGGAACAACATCGT	TGGCGGTCTTCATAGTAGCA
*G6Pase*	GATTCCGGTGTTTGAACGTC	GTAGAATCCAAGCGCGAAAC
*GPx1*	TTGGTGATTACTGGCTGCAC	CCATCTGAGGGGATTTTCCT
*GPx4*	GAGCCCATTCCTGAACCTTT	CGATGTCCTTGGCTGAGAAT
*HMOX1*	CAGAGCCGTCTCGAGCATAG	AAATCCTGGGGCATGCTGTC
*IL1β*	GCCCATCCTCTGTGACTCAT	AGGCCACAGGTATTTTGTCG
*IL6*	AAGCCAGAGTCCTTCAGAGAGA	GGAAATTGGGGTAGGAAGGA
*MCP-1*	CACTCACCTGCTGCTACTCATT	TCTGGACCCATTCCTTCTTG
*PEPCK*	AGCCTTTGGTCAACAACTGG	GTTATGCCCAGGATCAGCAT
*PPARα*	ATGCCAGTACTGCCGTTTTC	CCGAATCTTTCAGGTCGTGT
*PPARγ*	CAGGCCTCATGAAGAACCTT	ACCCTTGCATCCTTCACAAG
*SOD1*	CGGATGAAGAGAGGCATGTT	CACCTTTGCCCAAGTCATCT
*SOD2*	GCGGTCGTGTAAACCTCAAT	GATCTGCGCGTTAATGTGTG
*SREBP2*	AGAGGCGGACAACACACAAT	ACGCCAGACTTGTGCATCTT
*SREBP1c*	AAGAGCCCTGCACTTCTTGA	CCACAAAGAAACGGTGACCT
*TNFα*	TCTACTGAACTTCGGGGTGA	CACTTGGTGGTTTGCTACGA
*RPS29*	TCGTTGGGCGTCTGAAGGCAA	CGGAAGCACTGGCGGCACAT

Table 1. Sequences of primers used for real time PCR reactions. *ACC1*: Acetyl CoA carboxylase 1, *ACOX2*: Acetyl CoA oxidase 2, *CAT*: Catalase, *COL1A1*: Collagen 1A1, *CPT-1*: Carnitine palmitoyl transferase 1, *FAS*: Fatty acid synthase, *Gck*: Glucokinase, *G6Pase*: Glucose 6 phosphatase, *GPx1*: Glutathione peroxidase 1, *GPx4*: Glutathione peroxidase 4, *HMOX1*: Heme oxygenase 1, *IL1β*: Interleukine 1 beta, *IL6*: Interleukine 6, *MCP-1*: Macrophage chemoattractant protein 1, *PEPCK*: Phosphoenol pyruvate carboxykinase, *PPARα*: Peroxisome proliferator-activated receptor alpha, *PPARγ*: Peroxisome proliferator-activated receptor gamma, *SOD1*: Superoxide dismutase 1, *SOD2*: Superoxide dismutase 2, *SREBP2*: Sterol response element binding protein 2, *SREBP1c*: Sterol response element binding protein 1c, *TNFα*: Tumor necrosis factor alpha, and *RPS29*: Ribosomal Protein S29.
